# Potential preventive markers in the intracerebral hemorrhage process are revealed by serum untargeted metabolomics in mice using hypertensive cerebral microbleeds

**DOI:** 10.3389/fendo.2023.1084858

**Published:** 2023-04-20

**Authors:** Sai Wang, Xuelun Zou, Leiyun Wang, Huifang Zhou, Lianxu Wu, Yupeng Zhang, Tian-Xing Yao, Lei Chen, Ye Li, Yi- Zeng, Le Zhang

**Affiliations:** ^1^ Department of Neurology, Xiangya Hospital, Central South University, Changsha, Hunan, China; ^2^ Department of Pharmacy, Wuhan First Hospital, Wuhan, Hubei, China; ^3^ Department of Geriatrics, Second Xiangya Hospital, Central South University, Changsha, Hunan, China; ^4^ National Clinical Research Center for Geriatric Disorders, Xiangya Hospital, Central South University, Changsha, Hunan, China; ^5^ Multi-Modal Monitoring Technology for Severe Cerebrovascular Disease of Human Engineering Research Center, Xiangya Hospital, Central South University, Changsha, Hunan, China

**Keywords:** hypertensive intracerebral microbleeds, hypertension, metabolomics, biomarkers, liquid chromatography-mass spectrometry

## Abstract

Hypertensive cerebral microbleeds (HCMB) may be the early stage of hypertensive intracerebral hemorrhage (HICH), which is a serious threat to health due to its high mortality and disability rates. The early clinical symptoms of HCMB may not be significant. Moreover, it is difficult to achieve early diagnosis and intervention for targeted prevention of HICH. Although hypertension (HTN) is a predisposition for HCMB, it remains unclear whether there is any difference between hypertensive patients with or without HCMB. Therefore, we carried out liquid chromatography-mass spectrometry (LC-MS) to analyze early biomarkers for HCMB in mice with hypertension and to lay the foundation for early prevention of HICH in hypertensive patients. In total, 18 C57 male mice were randomly divided into the HCMB (n = 6), HTN (n = 6), and control groups (CON, n = 6). Hematoxylin-eosin and diaminobenzidine staining were used to assess the reliability of the model. The metabolite expression level and sample category stability were tested using the displacement test of orthogonal partial least squares discriminant analysis (OPLS-DA). Significant differences in metabolites were screened out using variable importance in the projection (VIP > 1), which were determined using the OPLS-DA model and the P-value of the t-test (*P* < 0.05) combined with the nonparametric rank-sum test. With an area under the curve (AUC) > 0.85 and a *P-*value of 0.05, the receiver operating characteristic curve (ROC) was used to further screen the distinct metabolites of HCMB. Compared with the HTN and CON groups, the HCMB group had significantly higher blood pressure and lower average body weight (*P* < 0.05). Through untargeted LC-MS analysis, 93 distinct metabolites were identified in the HCMB (*P* < 0.05, VIP > 1) group. Among these potential biomarkers, six significantly decreased and eight significantly increased differential metabolites were found. Meanwhile, we found that the HCMB group had statistically distinct arginine and purine metabolism pathways (*P* < 0.05), and citrulline may be the most significant possible biomarker of HCMB (AUC > 0.85, *P* < 0.05). All of these potential biomarkers may serve as early biomarkers for HICH in hypertension.

## 1 Introduction

Hypertension (HTN), which has a prevalence of more than 30%, has become a prevalent chronic disease owing to population aging and economic expansion. Because of its rapid onset, rapid progression, dramatic damage, high fatality, and disabling effects, intracerebral hemorrhage (ICH) is the most severe consequence of HTN and is also known as non-traumatic intracerebral hemorrhage (1–3). Hypertensive intracerebral hemorrhage (HICH), the most frequent modifiable independent risk factor for ICH, accounts for more than 50% of all ICH (2, 4–7) and has a higher risk of cerebral microbleeds (CMB) (1, 2, 8, 9). Even with the large-scale publicity on the importance of blood pressure control and the wide use of antihypertensive drugs, many patients with hypertension still take medication irregularly, their blood pressure is unstable, and cerebral vessels are still at risk of rupture due to rapid fluctuations in blood pressure (2, 6, 7, 10, 11). Studies on HTN and ICH revealed that CMB may be an intermediate state before HTN patients develop HICH, and it was found to predict the occurrence of ICH when combined with pathological analysis (1, 7, 12, 13).

However, because the majority of hypertensive cerebral microbleeds (HCMB) patients have no symptoms, imaging examinations have a low incidence of early detection (14, 15). Although hypertension is a predisposition to HCMB, it remains unclear whether there is any difference in patients with hypertension with or without HCMB. Therefore, exploring more avenues to uncover early biological indicators of HCMB may aid in determining the earliest stage of the disease. This could offer a specific direction for creating unique preventive plans for patients with HICH. However, there are insufficient precise and trustworthy biological indicators to warn people when HCMB is present, especially in patients with hypertension.

Metabolomics, which can flexibly connect genomes, transcriptomics, and proteomics and systematically monitor diverse metabolites at various stages and states of the life system, can screen biological indicators with high throughput (10, 16). Furthermore, metabolomics can study endogenous small molecules with molecular masses of less than 1000 (the molecular mass of the metabolite) (16) and has many benefits, including relatively small sample size requirements, a low number of differential metabolites, the potential for dynamic observation, easily understood results, and easy analysis when combined with phenotype and function (17–23). It also has a high likelihood of being used in the clinical setting.

In this study, we used untargeted metabolomics to provide an in-depth analysis of differential metabolites in normal, HTN, and HCMB mice to identify novel biomarkers for the early prevention of HICH and extend our understanding of HICH onset, progression, and prognostic mechanisms.

## 2 Methods

### 2.1 Animal models construction

The animal experiments in this study were approved by the Ethics Committee of the Department of Laboratory Animal Science at Central South University and were conducted according to the National Institutes of Health guidelines for the care and use of laboratory animals. Additionally, the manuscript followed the ARRIVE Animal Research: Reporting of Experiments in Vivo (ARRIVE) Guide for Laboratory Animals (24). The trials were performed in the Department of Laboratory Animal Science, Xiangya School of Medicine, Central South University, using SPF-grade C57BL6/J mice, whose age and weight were restricted to 30–32 weeks and 28–32 g, respectively. Female mice were excluded from this study because of their susceptibility to steroid hormones. The mice were acquired from Beijing HFK Bio-Technology Co., LTD and housed in the Department of Zoology’s SPF-level Laboratory Animal Room (room temperature maintained at 23.0°C, humidity at 60–70%, and artificial circadian lighting for 12 h). Each male mouse was maintained in a single cage to avoid aggression.

**Figure 1:**
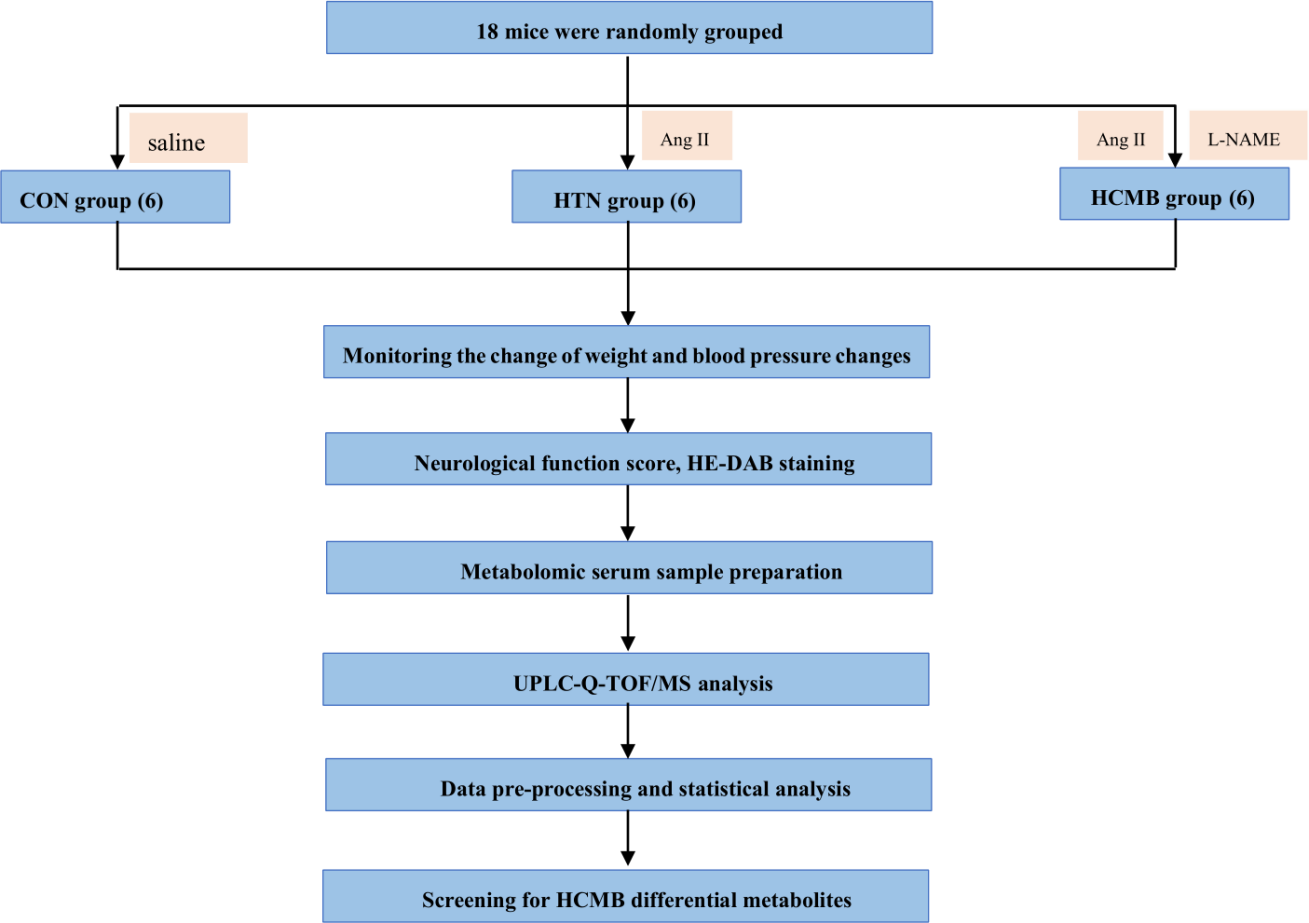
Flowchart of the study design. CON, control group; HTN, hypertension; HCMB, hypertensive cerebral microbleeds; Ang II, angiotensin II; L-NAME, levonitroarginine methyl ester; HE-DAB, Hematoxylin eosin staining and diaminobenzidine staining; UPLC-Q-TOF-MS, ultraperformance liquid chromatography coupled with quadrupole time-of-flight mass spectrometry technique.

**Figure 2:**
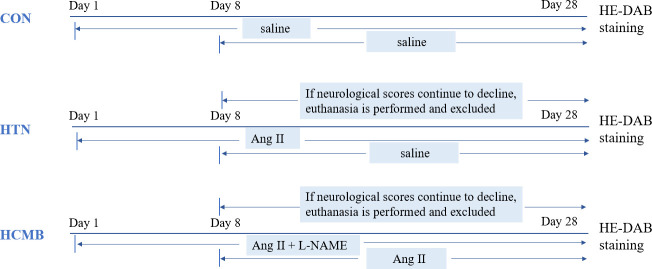
Diagram of experimental modeling process. CON, control group; HTN, hypertension; HCMB, hypertensive cerebral microbleeds; Ang II, angiotensin II; L-NAME, levonitroarginine methyl ester; HE-DAB, Hematoxylin eosin staining and diaminobenzidine staining.

**Figure 1** shows the experimental design. Using the SPSS random number generator function, 18 male C57BL6/J mice were randomly assigned according to their body weight (CON = 6, HTN = 6, and HCMB = 6), and the average body weight of each group was 31 g. Next, the right ear of each experimental mouse was marked using ear-tagging forceps so that each group of mice could be distinguished. Before the formal experiment, all mice were acclimated to the Department of Zoology’s barrier environment for 10 days, and mice were trained in the non-invasive tail vein blood pressure monitor to ensure accurate blood pressure measurements and initial blood pressure measurements. We used a non-invasive sphygmomanometer (IITC Life Sciences) to measure blood pressure in mice (25, 26). Blood pressure measurements were conducted by a blinded researcher. The mice underwent blood pressure measurement training 1 week prior to the first official measurement to ensure they were comfortable with the environment and process. The fixators (Mouse Fixator, Medium (for fixing 25-50g mice)) are available for blood pressure measurement. Furthermore, when measuring blood pressure using the tail cuff method, the mice were given time to stabilize to avoid irritation and movement. During measurements, blood flow to the tail was blocked through an airbag placed at the base of the tail, which was then deflated gradually, while a piezoelectric pulse sensor was placed in the tail of the mice and blood pressure measured using a combination of the two. As shown in **Figure 2**, in addition to the implantation of an extended-release 1000ng/kg/day Ang II capsule pump (day 1 to day 28), Ang II (0.5μg/g, twice daily) was injected subcutaneously into the HCMB group from days 8 to 28 of the modeling (24, 25, 27–29) (**Figure 2**). In addition, L-NAME, which was used to induce HCMB, was only used in the HCMB group from days 1 to 28 and was added to drinking water at a dose of 100 mg/kg/day (24, 25, 27–29). During the experiment, if we found that some mice showed signs of reduced neurological function, we performed MRI on the mice with neurological function scores below 16 (according to the modified Garcia score) (30, 31) and excluded them if they were considered to have ICH. Prior to the formal blood pressure measurement, we anesthetized the mice using barbiturates, immobilized them on the MRI instrument after they were better anesthetized, and then performed MRI measurements. The heads of the mice were scanned with small animal MRI (BS-70) (BioSpec70/20USR), adjusting the position of the head so that the center area of the scan was at the level of the potential bleeding area. The state of the model was confirmed by hematoxylin-eosin and diaminobenzidine staining. Neurological function scores, MRI, and HE-DAB staining were performed by blinded investigators.

### 2.2 Mice terminating

The orbital blood collection method was utilized after the mice were anesthetized with a barbiturate anesthetic. An abdomen incision was then made to expose the abdominal organs, and the diaphragm was cut to fully expose the heart. The needle was then inserted along the tip of the heart, and the mice were perfused with sterile saline; the perfusion was stopped when the liver and kidneys turned white. Finally, the skull was cut open with a pair of scissors, and the brain tissue was collected after separating the skull with forceps. The brain tissue was placed in formalin for fixation.

### 2.3 Histological processing

The hematoxylin-eosin staining process began with a sample that was previously fixed in formaldehyde. Next, the tissue was rinsed, and the water was gradually removed from the block using a low-high concentration of alcohol as a dehydrating agent. The tissue block was then placed in xylene, a transparent agent dissolved in both alcohol and paraffin, to replace the alcohol in the block before embedding it in wax. Thereafter, the transparent tissue block was placed in melted paraffin wax and a waxing chamber for insulation. After the tissue block was completely immersed in the wax, the block was embedded, cooled, and solidified. The embedded wax block was fixed on a slicer and cut into thin slices, usually 5–8 µm thick. Before staining, the paraffin was removed from the sections using xylene. The tissue slices were then stained with hematoxylin aqueous solution and alcoholic eosin stain *via* a high-low concentration of alcohol, and finally distilled water. Some sections were stained using hematoxylin-eosin combined with diaminobenzidine (DAB) to highlight the presence of hemorrhage. The B stain (1:25) was diluted with distilled water as a working solution before use, and then the A stain was added in proportion (1:25), mixed, and used immediately. The color development time was 1–30 minutes; the color development reaction was terminated by rinsing with running water. When DAB reacts with the peroxidase enzyme present in red blood cells, it turns brown, thus allowing the precise detection of extravasated blood cells in the brain parenchyma (25).

### 2.4 Sample preparation

Following the initial evaluation of neurological function, the mice were sedated with pentobarbital anesthesia. Orbital venous blood was collected by removing the eyeball, and after 30 min of resting, the blood was centrifuged at 3000 rpm for 10 min. The serum was then collected and frozen at –80°C for future investigations.

### 2.5 Metabolomics samples processing by liquid chromatography-mass spectrometry

Each group of six samples was placed on ice after defrosting. First, we carefully pipetted 100 µL of the sample into a 1.5 mL centrifuge tube and added 400 L of extraction solution containing 0.02 mg/ml of internal standard (methanol: acetonitrile = 1:1 (v/v)) (L-2-chlorophenylalanine). Second, we vortex and mixed for 30 s, then extracted using low-temperature sonication (5°C, 40 kHz) for 30°min. Third, hold the sample at –20°C for 30°min. Fourth, Centrifuge for 15°min (13,000 g, 4°C), remove the supernatant and dry the sample under nitrogen. Fifth, to re-dissolve the samples, 100 µL of the compound solution (acetonitrile: water = 1:1) was added. Sixth, the samples were vortexed and mixed for 30 s, followed by 5°min of low-temperature ultrasonic extraction (5°C, 40 kHz). Finally, centrifuge (Eppendorf GmbH, Germany) for 10 minutes at 13,000 g and 4°C, then transfer the supernatant to the injection vial with an internal cannula for analysis. In addition, 20 μL of each sample’s supernatant was pipetted separately and combined as a quality control sample.

The Ultra-High Performance Liquid Chromatography-Tandem Fourier Transform Mass Spectrometry UHPLC -Q Exactive system (Thermo Fisher) was used as the instrument platform for this LC-MS investigation. The chromatographic and mass spectrometric studies that were employed were as follows: 2 μL of the sample was separated on an HSS T3 column (100 mm 2.1 mm i.d., 1.8 m) for chromatographic analysis, and then proceeded to mass spectrometric detection. Mobile phase B was composed of 47.5% acetonitrile, 47.5% isopropanol, and 5% water (containing 0.1% formic acid), whereas mobile phase A was composed of 95% water and 5% acetonitrile (containing 0.1% formic acid). The separation gradient was as follows: from 0 to 0.1 min, mobile phase B linearity increased from 0% to 5%; from 5% to 25%; from 1–2 min; from 2–9 min, the linearity of mobile phase B increased from 25% to 100%; mobile phase B maintained 100% linearity between 9 and 13 min; between 13.0 and 13.1 minutes, dropping to 0% linearity. For 13.1-16 min, the linearity of mobile phase B was maintained at 0%. The column temperature was 40°C and the flow rate was 0.40 mL/min. The sample mass spectrometer signal acquisition was performed in positive and negative ion scan modes with a mass scan range of m/z:70–1050 for mass spectrometry analysis. Ion source heating temperature, sheath gas pressure, auxiliary heating gas pressure, cyclic collision energy, MS1 resolution, and MS2 resolution were 400°C, 40 psi, 10 psi, 20–40–60 V, 70,000, and 17500, respectively. Metabolomics studies were also carried out by blinded investigators.

### 2.6 Data pre-processing and library search

The mass spectrometry data were loaded into Progenesis QI 2.3 (Waters Corporation, Milford, USA), a program for processing metabolomics data, for preliminary processing. After baseline filtering, peak identification, integration, retention time correction, peak alignment, and finally, a data matrix of retention time, mass-to-charge ratio, and peak intensity was calculated based on the data. To identify the metabolites, the HMDB (http://www.hmdb.ca/) and Metlin (https://metlin.scripps.edu/) metabolic public databases and Meguiar’s library (32) were compared with the MS and MSMS mass spectra.

For the data analysis, the searched matrix data were uploaded to Meguiar’s BioCloud platform (cloud.majorbio.com). The data matrix was first preprocessed to remove missing values using the 80% rule, which entails keeping at least one set of variables with non-zero values above 80%. Next, the gaps were filled using the smallest value from the original matrix, and the response intensities of the sample mass spectrometry peaks were normalized using sum normalization to reduce errors caused by sample preparation and instrument instability. This resulted in a normalized data matrix. To create the final data matrix for further analysis, variables with a relative standard deviation (RSD) >30% of the QC samples were eliminated and log10 logged.

### 2.7 Differential metabolite analysis and related statistical analysis

The variance in the pre-processed matrix files was examined. Principal component analysis (PCA) and orthogonal least squares discriminant analysis (OPLS-DA) were carried out using ropls (Version 1.6.2), and seven rounds of cross-validation were employed to evaluate the model’s stability. Also carried out were the student’s t-test and the multiplicative analysis of variance. Based on the student’s t-test *P-*values and variable weight values (VIPs) derived from the OPLS-DA model, differential metabolites were chosen. The differential metabolites were selected using the unidimensional test threshold *P* < 0.05 and the intersection of multidimensional statistics of variable importance in the projection (VIP) >1. Finally, 272 distinct metabolites in all were examined.

The raw data was analyzed using the metabolome search software ProgenesisQI 2.0, and the metabolites were identified using primary mass spectrometry (MS1) with a precision molecular weight mass error range of 10 PPm, MS2 (secondary mass spectrometry) with secondary identification and isotopic distribution patterns, so the compounds were screened at the level 2 of the Sumner et al. (2007) criteria. level2 level. Fold change (FC) was employed to determine the differential expression of metabolites. Using the Kyoto encyclopedia of genes and genomes (KEGG) database (https://www.kegg.jp/kegg/pathway.html), the differential metabolites were mapped to metabolic enrichment and pathway analysis, and topological analysis was used to calculate the route influencing extent. The results were displayed as the mean standard deviation. The Fisher’s exact test was utilized to identify the most pertinent biologic pathways, and the scipy.stats (Version 1.0.0) was employed for pathway enrichment analysis. Groups were compared using the nonparametric rank-sum test and the student’s t-test, with a significance level of *P* < 0.05. In addition, the data of differential metabolites screened from blood samples were imported into the SPSS 26.0 program and the subject operating characteristic (ROC) curve was generated to screen and validate the illness discriminatory ability of differential metabolites. The elimination of potential biological markers based on an area under the curve (AUC) greater than 0.85. Software information relevant to the analysis of this study is detailed in **Supplementary Table 1**.

## 3 Results

### 3.1 HTN and HCMB mouse models were constructed successfully

**Figure 3:**
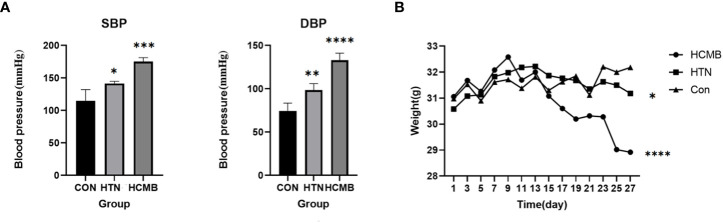
Plots of blood pressure **(A)** and body weight **(B)** of the modeled mice over time. The vertical coordinate of figure **(A)** is blood pressure, and the horizontal coordinates are for CON (n = 6), HTN (n = 6) and HCMB (n = 6) groups. Figure **(B)**: Vertical coordinate is weight (/g), horizontal coordinate is experimental day. Note: From the third day of the experiment, there was a statistically significant difference between the HCMB group and the HTN and CON groups (p<0.05), and on the 13th day, there was a statistically significant difference in weight (p<0.001). “*” represent p<0.05,“**” represent p<0.01,“***” represent p<0.001,“****” represent p<0.0001.

Following subcutaneous implantation of capsule pumps and ingestion of L-NAME, the mice were regularly monitored for body weight (**Figure 3A**), blood pressure (**Figure 3B**), and neurological function score.

**Figure 4:**
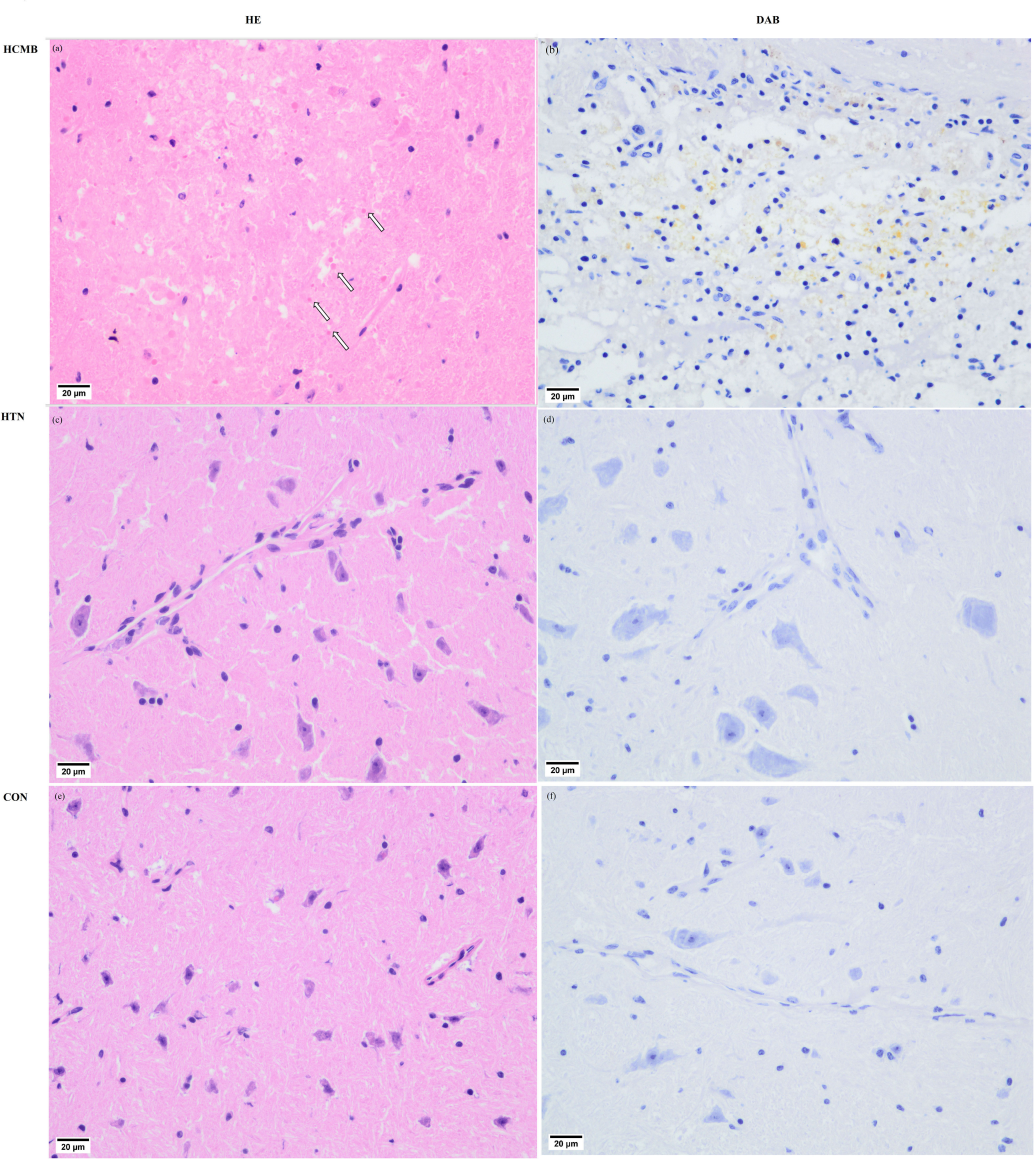
Pathological images of cerebral microbleeds. **(A)** Hematocrit enrichment map of hypertensive cerebral microbleeds tissue. In the ICH, blood cells are evident where the HE-stained arrows point in the diagram, and **(B)** DAB-stained blood cells (pale yellow) are visible in the diagram. diaminobenzidine staining images of hypertensive cerebral microbleeds. **(C)** HE stained picture of brain tissue in the hypertensive group. **(D)** DAB staining of brain tissue in the hypertensive group. **(E)** HE staining map of brain tissue in the control group. **(F)** DAB staining map of brain tissue in the control group.

In the HCMB group, Ang II injections were initiated on the eighth day of modeling. Because the noninvasive tail vein blood pressure monitor is limited and congested, blood pressure was not measured immediately following administration. In addition, continuous blood pressure monitoring may cause stroke, and Ang II-induced blood pressure variations in mice have been reported previously (24, 33). The blood pressure of HCMB mice was substantially higher than that of HTN and CON mice following 28 days of monitoring (*P* < 0.05). In addition to blood pressure abnormalities, the mice in the HCMB group exhibited considerable wasting. Mice had decreased food and water intake, decreased urine production, and nearly no feces. The behavior of the mice in the HTN group was normal, and there were no significant alterations in the CON group. Body weight was significantly lower in the HCMB group than in the HTN and CON groups (*P* < 0.05). The 3.0T MRI sequence on day 28 of the modeling exclusion of ICH and the histological brain slice confirmed the presence of microhemorrhage (**Figure 4**). All mice were excluded because they did not meet the needs of the experiment and were handled in accordance with the ethical code of the Department of Laboratory Animal Science of Central South University.

### 3.2 Different metabolites profiles were observed in HCMB mice

**Figure 5:**
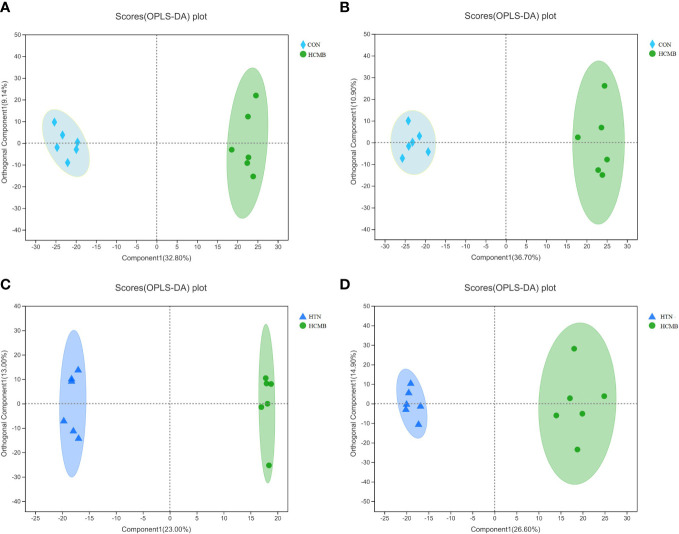
OPLS-DA score plot. **(A)**: HCMB vs CON cation table scatter plot. **(B)**: HCMB vs CON anion table scatter plot. **(C)**: HCMB vs HTN cation table scatter plot. **(D)**: HCMB vs HTN anion scatter plot. Comp1 first predicted principal component explanatory degree, orthogonal Comp1 first orthogonal component explanatory degree. The greater the separation of the two groups of samples in the graph, the more significant the classification effect. OPLS-DA, orthogonal least partial square discriminant analysis; CON, control group; HTN, hypertension; HCMB, hypertensive cerebral microbleeds.

**Figure 6:**
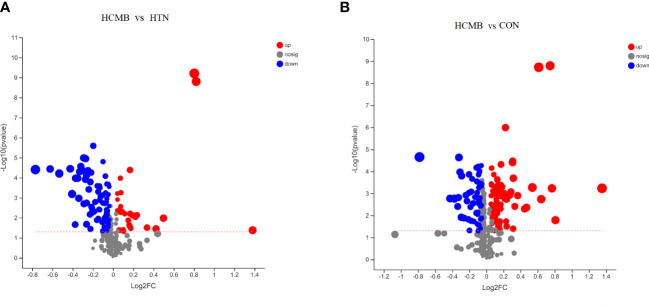
Volcano plots of the differences between groups. **(A)**. Volcano plots of intergroup differences between the two groups of HCMB vs HTN. **(B)**. Volcano plot of intergroup differences between two groups of HCMB vs CON. The horizontal coordinate of the graph is the value of the fold change in metabolite expression difference between the two groups, i.e. log2FC, and the vertical coordinate is the value of the statistical test for the difference in metabolite expression change, i.e. -log10(p_value), the higher the value the more significant the expression difference, the values of the horizontal and vertical coordinates have been logarithmicized. Each point in the graph represents a specific metabolite and the size of the point indicates the Vip value. The points on the left are metabolites with down-regulated expression differences and the points on the right are metabolites with up-regulated expression differences, with the more significant expression differences occurring to the left, right and top of the graph. CON, control group; HTN, hypertension; HCMB, hypertensive cerebral microbleeds.

Three-dimensional PCA scatter plots were used to evaluate the overall differences between the groups of samples and the degree of variability across samples within groups. Serum samples from the CON, HTN, and HCMB groups were separated and both positive and negative ion mode principal component score plots were generated. In addition to PCA, OPLS-DA was employed for multivariate analysis to exclude factors in the X data variables that were unrelated or orthogonal to the Y variables. This enhanced the model’s validity and resolution by allowing for a more precise distinction between intergroup differences. As illustrated in **Figures 5**, **6**, the three groups were divided and clustered, indicating that their serum metabolic profiles were distinct. According to the OPLS-DA replacement test results, R^2^Y(cum) _(+)_ = 0.998, Q^2^_(+)_ = 0.828, R^2^Y(cum) _(–)_ = 0.983, Q^2^_(-)_ = 0.845 in the HCMB and HTN groups; R^2^Y(cum) _(+)_ = 0.992, Q^2^_(+)_ = 0.829, R^2^Y(cum) _(–)_ = 0.991, Q^2^_(–)_ = 0.901 in the HCMB and CON groups; R^2^Y(cum) _(+)_ = 0.985, Q^2^_(+)_ = 0.683, R^2^Y(cum) _(–)_ = 0.999, Q^2^_(–)_ = 0.801 in the HTN and CON groups. R^2^Y, R^2^Y(cum), and Q^2^ were greater than 0.5, and close to 1 across the three groups, demonstrating a reasonable fit and predictive capacity of the model.

### 3.3 272 Potential biomarkers of HCMB were identified

**Figure 7:**
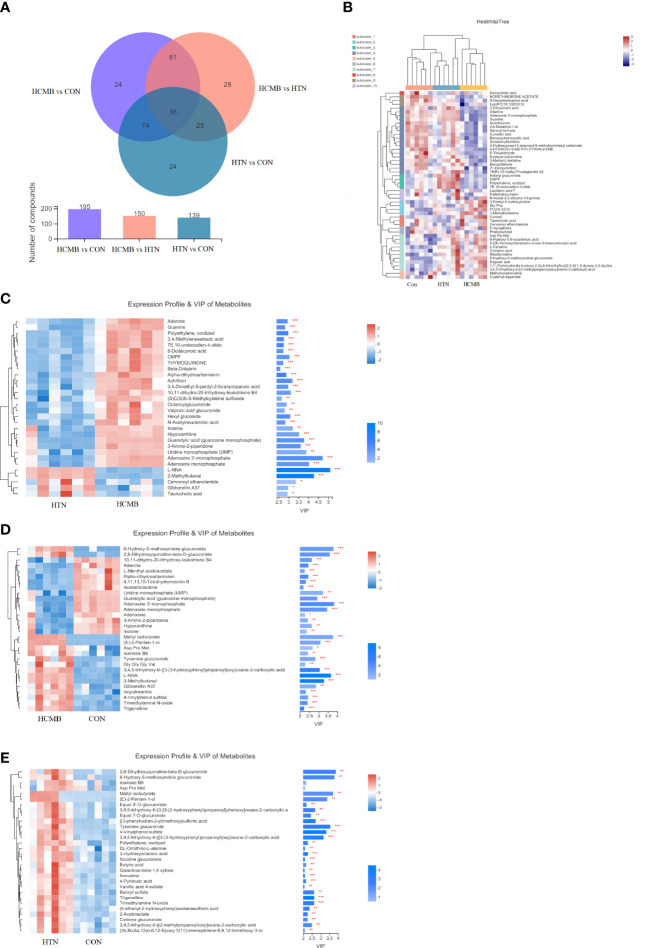
Venn diagram and heat map of HCMB with HTN and CON groups. **(A)**. Venn diagram of differential metabolites between HCMB vs CON groups. Note: The overlapping parts of the graph indicate the number of metabolites common to multiple metabolic sets, the parts that do not overlap indicate the number of metabolites specific to that metabolic set, and the numbers indicate the corresponding metabolite numbers. The second bar graph indicates the number of metabolites contained in each metabolite set. The third graph is used to depict the number of metabolites contained in each part of the Venn diagram, the number of intersecting parts and the number of difference parts. **(B)**. Heat map of differential VIP values between HCMB, HTN and CON groups. In the graph, each row denotes a metabolite and each column a sample. The numbers under the color bar at the bottom right of the graph represent the trend in expression, and the colors in the graph show the relative expression of the metabolite in that group of samples. The name of the metabolite is on the right, and on the left is a tree diagram of metabolite clustering. The closer two metabolite branches are to one another, the closer their expressions are. The names of the samples are displayed on the lower side, and the upper side displays a tree diagram of sample clustering. The expression patterns of all metabolites in these two samples are more closely correlated with one another the closer two sample branches are to one another. **(C)**. Clustering diagram of differential metabolites between HCMB vs HTN groups. **(D)**. Clustering diagram of differential metabolites between HCMB vs CON groups. **(E)**. Clustering diagram of differential metabolites between HTN vs CON groups. **(C-E)** Each row represents a metabolite, the color indicating the size of the relative expression of that metabolite in that group of samples, and the color gradient corresponds to the size of the value is shown in the gradient color block. The left side is a metabolite clustering tree; the closer the branches, the closer the expression pattern of all metabolites within the sample. Each column represents a sample, and the sample name is below. The metabolite VIP bar is located on the right side; its length indicates how much the metabolite contributed to the difference between the two groups; its default value is not less than 1, and a larger value indicates that the metabolite contributed more to the difference between the groups. The P value, which is represented by the bar’s color, reflects the importance of the metabolite difference between the two groups. The deeper the color, the smaller the P value, and the bigger the -log10(P-value). Right side * denotes P<0.05, ** denotes P<0.01, and *** denotes P<0.001. CON, control group; HTN, hypertension; HCMB, hypertensive cerebral microbleeds.

**Figure 8:**
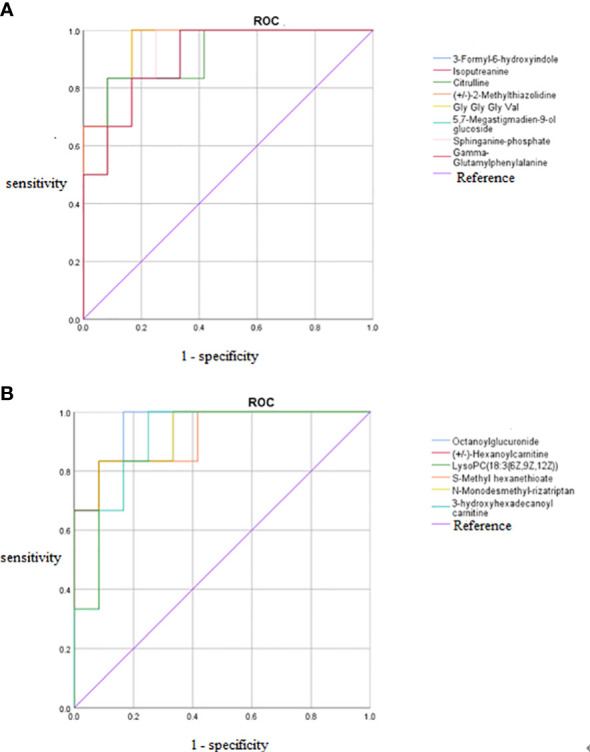
ROC curves of 14 potential biomarkers. **(A)** Increased potential biomarker ROC curves, **(B)** Decreased potential biomarker ROC curves. The AUC represents the area under the ROC curve and indicates the prediction accuracy. the AUC value takes on a value between 0 and 1, with larger values representing higher correctness rates. ROC, receiver operating characteristic curve; AUC, area under the curve.

In total, 272 differential metabolites (**Supplementary Table 2**) were screened by setting the VIP > 1 combined with *P* < 0.05 in the OPLS-DA method, among which 195 and 150 differential metabolites were found in the HCMB vs. CON group and HCMB vs. HTN group, respectively (**Figure 7A**). Furthermore, a total of 97 differential metabolites (32 increased and 65 decreased) were identified in the HCMB group, which were different from those in the CON and HTN groups. As shown in **Figure 8**, among the 65 decreased metabolites, four differential metabolites, 5methylpyrazine-2-carboxylic acid, PE (18:3/0:0), inosine, and adenosine did not meet the criteria of the rank-sum test and were excluded (**Tables 1**, **2**). The top 50 specifically expressed metabolites in the CON, HTN, and HCMB groups of mice are displayed in the clustering heat map (**Figure 7B**). Metabolites with similar abundance trends are expressed in close proximity. The expression of the differential metabolites in the three groups showed significant intergroup differences. This is because the metabolites in the HCMB group were significantly clustered together and separated from the metabolites of the other groups. To visualize the importance and changes in expression trends of differential metabolites in the two groups (HCMB vs. HTN, HCMB vs. CON, and HTN vs. CON), the unidimensional statistics of *P-*values and multivariate statistical analysis of VIP are presented in **Figures 7C-E**.

**Table 1:** LC-MS analysis of metabolites upregulated in the HCMB group compared to the HTN and Con groups by the rank-sum test.

	Metabolite	HCMB		HTN		Con		P value	FDR	FC		Mode
		mean	SD	mean	SD	mean	SD			HCMB/ Con	HCMB/ HTN	
1	3-Formyl-6-hydroxyindole	3.660	0.281	3.269	0.167	3.088	0.185	0.007	0.032	1.185	1.120	–
2	Atractyligenin	3.798	0.503	3.015	0.279	2.941	0.359	0.014	0.047	1.291	1.260	–
3	2-Keto-6-acetamidocaproate	5.201	0.084	4.950	0.054	4.935	0.108	0.003	0.048	1.054	1.051	+
4	LysoPC(P-18:0)	5.505	0.063	5.344	0.061	5.282	0.084	0.004	0.048	1.042	1.030	+
5	PC (17:0/0:0)	6.152	0.064	5.973	0.062	5.896	0.072	0.002	0.048	1.043	1.030	+
6	PS(O-20:0/0:0)	4.730	0.101	4.483	0.067	4.452	0.106	0.003	0.048	1.062	1.055	+
7	PE (22:1/0:0)	5.034	0.182	4.733	0.093	4.615	0.107	0.002	0.048	1.091	1.064	+
8	2-O-Methylcytosine	4.787	0.175	4.264	0.062	4.252	0.083	0.003	0.048	1.126	1.123	+
9	Ganosporeric acid A	4.454	0.380	3.893	0.162	4.055	0.086	0.006	0.048	1.098	1.144	+
10	PC (19:0/0:0)	5.156	0.074	5.017	0.055	4.953	0.059	0.005	0.048	1.041	1.028	+
11	2-Methylbutanal	3.967	0.172	2.244	0.110	2.366	0.081	0.001	0.048	1.677	1.768	+
12	L-NNA	5.730	0.166	3.283	0.205	3.747	0.172	0.001	0.048	1.529	1.745	+
13	PC (20:2/0:0)	5.733	0.184	5.449	0.068	5.247	0.107	0.001	0.048	1.093	1.052	+
14	PC (22:4/0:0)	4.969	0.215	4.754	0.082	4.523	0.094	0.002	0.048	1.099	1.045	+
15	LysoPE (20:3(5Z,8Z,11Z)/0:0)	4.765	0.157	4.587	0.094	4.348	0.129	0.002	0.048	1.096	1.039	+
16	Asp Ala	3.790	0.381	3.254	0.145	2.942	0.271	0.002	0.048	1.288	1.165	+
17	Isoputreanine	4.544	0.182	4.198	0.165	3.651	0.339	0.001	0.048	1.245	1.082	+
18	Citrulline	4.363	0.162	4.182	0.079	4.052	0.152	0.016	0.050	1.077	1.043	–
19	Gly Gly Gly Val	3.966	0.696	3.144	0.401	2.908	0.199	0.008	0.051	1.364	1.261	+
20	THTC	5.255	0.123	5.033	0.079	5.012	0.119	0.008	0.051	1.048	1.044	+
21	(+/-)-2-Methylthiazolidine	4.536	0.113	4.308	0.085	4.304	0.122	0.008	0.051	1.054	1.053	+
22	4-Hydroxyretinoic acid	4.311	0.417	3.658	0.294	3.597	0.244	0.018	0.053	1.198	1.179	–
23	Ser Ile Ala Asp	4.856	0.345	4.319	0.163	4.178	0.187	0.010	0.054	1.162	1.124	+
24	Sphinganine-phosphate	4.662	0.124	4.486	0.142	4.369	0.148	0.011	0.055	1.067	1.039	+
25	5,7-Megastigmadien-9-ol glucoside	4.355	0.321	3.900	0.260	3.714	0.178	0.011	0.055	1.173	1.117	+
26	Gibberellin A37	4.073	0.650	2.884	0.661	2.611	0.550	0.015	0.061	1.560	1.412	+
27	Gamma-Glutamylphenylalanine	4.357	0.164	4.042	0.232	4.009	0.181	0.023	0.063	1.087	1.078	–
28	Glu Thr	4.155	0.238	3.875	0.181	3.701	0.199	0.022	0.072	1.123	1.072	+
29	L-Ornithine	3.261	0.366	2.889	0.056	2.779	0.207	0.025	0.077	1.173	1.129	+
30	Glu Phe	4.743	0.168	4.495	0.197	4.449	0.145	0.026	0.078	1.066	1.055	+
31	{[1-hydroxy-1-(1-oxo-1H-isochromen -3-yl)but-3-en-2-yl]oxy}sulfonic acid	4.638	0.310	4.328	0.080	4.337	0.111	0.029	0.085	1.069	1.072	+
32	Cortisol	5.009	0.405	4.470	0.303	4.488	0.207	0.049	0.116	1.116	1.121	+

HCMB, hypertensive intracerebral hemorrhage; HTN, hypertension; CON, control group; SD, standard deviation; FDR, false discovery rate. Mode: ion detection modes, including positive (+) and negative (-) ion modes.

**Table 2:** LC-MS analysis of metabolites downregulated in the HCMB group compared to the HTN and Con groups by the rank-sum test.

	Metabolite	HCMB		HTN		Con		P value	FDR	FC		Mode	
		mean	SD	mean	SD	mean	SD			HCMB/ Con	HCMB/ HTN		
1	4,11,13,15-Tetrahydroridentin B	3.554	0.148	4.278	0.219	4.545	0.320	0.002	0.027	0.782	0.831	–	
2	Guanidylic acid (guanosine monophosphate)	2.983	0.670	4.842	0.129	4.705	0.148	0.002	0.027	0.634	0.616	–	
3	2,2'-(3-methylcyclohexane-1,1-diyl)diacetic acid	3.516	0.105	3.994	0.215	3.967	0.261	0.003	0.027	0.886	0.880	–	
4	Alpha-dihydroartemisinin	3.008	0.185	4.010	0.221	4.104	0.307	0.003	0.027	0.733	0.750	–	
5	C75	3.863	0.099	4.299	0.176	4.343	0.259	0.003	0.027	0.889	0.899	–	
6	5-Nonyltetrahydro-2-oxo-3-furancarboxylic acid	3.741	0.068	4.138	0.188	4.251	0.351	0.003	0.027	0.880	0.904	–	
7	10-Oxo-11-octadecen-13-olide	3.248	0.148	3.637	0.212	3.690	0.127	0.004	0.027	0.880	0.893	–	
8	10,11-dihydro-20-trihydroxy-leukotriene B4	3.453	0.491	4.644	0.201	4.762	0.188	0.003	0.027	0.725	0.744	–	
9	L-Menthyl acetoacetate	2.351	0.111	3.104	0.260	3.354	0.487	0.003	0.027	0.701	0.757	–	
10	3,4-Dimethyl-5-pentyl-2-furanpropanoic acid	2.702	0.377	3.831	0.269	3.514	0.358	0.002	0.027	0.769	0.705	–	
11	Riesling acetal	3.019	0.148	3.705	0.157	3.730	0.330	0.003	0.027	0.809	0.815	–	
12	Adenosine monophosphate	2.971	0.863	5.246	0.342	5.323	0.278	0.003	0.027	0.558	0.566	–	
13	2-Hydroxydecanedioic acid	3.718	0.206	4.408	0.186	4.044	0.158	0.001	0.027	0.919	0.843	–	
14	N-Acetylneuraminic acid	2.589	0.273	3.649	0.331	3.105	0.316	0.002	0.027	0.834	0.710	–	
15	Valproic acid glucuronide	3.444	0.434	4.516	0.414	4.131	0.202	0.004	0.028	0.834	0.763	–	
16	Uridine monophosphate (UMP)	2.372	1.438	4.789	0.187	4.744	0.151	0.004	0.028	0.500	0.495	–	
17	LysoPC(18:3(6Z,9Z,12Z))	4.739	0.288	5.084	0.108	5.181	0.068	0.006	0.031	0.915	0.932	–	
18	13(S)-HODE	4.446	0.126	4.755	0.104	4.749	0.155	0.006	0.031	0.936	0.935	–	
19	S-Methyl hexanethioate	3.311	0.424	4.241	0.348	3.767	0.268	0.009	0.035	0.879	0.781	–	
20	Adenine	3.801	0.160	4.688	0.273	4.749	0.270	0.003	0.048	0.800	0.811	+	
21	3,4-Methylenesebacic acid	3.089	0.141	3.857	0.217	3.542	0.473	0.004	0.048	0.872	0.801	+	
22	Monoethylhexyl phthalic acid	4.319	0.112	4.528	0.093	4.593	0.074	0.004	0.048	0.940	0.954	+	
23	Traumatic acid	3.853	0.077	4.029	0.057	4.154	0.140	0.002	0.048	0.928	0.956	+	
24	Adenosine 3'-monophosphate	3.264	0.727	5.565	0.358	5.615	0.268	0.003	0.048	0.581	0.587	+	
25	3-Amino-2-piperidone	3.040	0.479	4.400	0.162	4.083	0.364	0.005	0.048	0.745	0.691	+	
26	Thymine	4.486	0.086	4.689	0.102	4.748	0.134	0.005	0.048	0.945	0.957	+	
27	ACETYL-L-CYSTEINE	3.200	0.130	3.700	0.293	3.641	0.262	0.006	0.048	0.879	0.865	+	
28	Niacinamide	5.303	0.075	5.542	0.071	5.528	0.073	0.003	0.048	0.959	0.957	+	
29	Guanine	3.186	0.253	4.012	0.159	3.893	0.152	0.003	0.048	0.818	0.794	+	
30	Mevalonolactone	4.022	0.250	4.431	0.098	4.476	0.132	0.006	0.048	0.899	0.908	+	
31	Achillicin	2.993	0.234	4.019	0.270	3.752	0.362	0.003	0.048	0.798	0.745	+	
32	Palmitoyl-L-carnitine	6.059	0.042	6.227	0.063	6.303	0.109	0.003	0.048	0.961	0.973	+	
33	Benzoquinoneacetic acid	4.115	0.086	4.314	0.098	4.384	0.069	0.003	0.048	0.939	0.954	+	
34	(+/-)-Myristoylcarnitine	5.644	0.081	5.834	0.053	5.920	0.161	0.004	0.048	0.953	0.967	+	
35	Isoalantolactone	3.288	0.095	3.826	0.173	4.072	0.297	0.002	0.048	0.807	0.859	+	
36	P-Tolualdehyde	3.049	0.061	3.339	0.118	3.516	0.240	0.002	0.048	0.867	0.913	+	
37	Dodecanoylcarnitine	5.024	0.075	5.226	0.122	5.316	0.112	0.003	0.048	0.945	0.961	+	
38	5,7,9,11,13-tetradecapentaenoic acid	3.902	0.068	4.189	0.130	4.235	0.234	0.005	0.048	0.921	0.931	+	
39	3-hydroxydodecanoyl carnitine	4.771	0.061	5.025	0.110	5.150	0.159	0.003	0.048	0.926	0.949	+	
40	Parthenolide	2.923	0.086	3.259	0.122	3.689	0.496	0.001	0.048	0.792	0.897	+	
41	9Z,13-Tetradecadien-11-ynal	3.227	0.176	3.834	0.126	3.812	0.268	0.003	0.048	0.847	0.842	+	
42	Norecasantalic acid	3.253	0.068	3.729	0.102	3.744	0.189	0.003	0.048	0.869	0.872	+	
43	Cyclohexanone	3.347	0.205	3.672	0.060	3.787	0.101	0.001	0.048	0.884	0.911	+	
44	(+/-)-Hexanoylcarnitine	4.795	0.165	4.986	0.065	5.106	0.086	0.005	0.048	0.939	0.962	+	
45	Polyethylene, oxidized	3.929	0.145	4.808	0.222	4.202	0.262	0.002	0.048	0.935	0.817	+	
46	7E,10-undecadien-4-olide	3.632	0.127	4.385	0.191	3.949	0.198	0.001	0.048	0.920	0.828	+	
47	4-hydroxy-6-methylpyran-2-one	4.345	0.076	4.549	0.089	4.711	0.115	0.001	0.048	0.922	0.955	+	
48	Octanoylglucuronide	3.457	0.263	4.376	0.419	3.830	0.192	0.002	0.048	0.903	0.790	+	
49	8-Dodecynoic acid	2.781	0.100	3.543	0.286	3.121	0.109	0.001	0.048	0.891	0.785	+	
50	N-(3-oxo-octanoyl)-homoserine lactone	3.460	0.110	4.089	0.273	3.704	0.139	0.001	0.048	0.934	0.846	+	
51	(S)C(S)S-S-Methylcysteine sulfoxide	3.644	0.448	4.453	0.169	4.213	0.158	0.002	0.048	0.865	0.818	+	
52	METHACHOLINE	5.780	0.081	6.096	0.091	6.023	0.134	0.007	0.050	0.960	0.948	+	
53	2-Methylfuran	3.444	0.108	3.784	0.106	3.698	0.159	0.007	0.051	0.931	0.910	+	
54	7-Hydroxy-6-(methoxyacetyl)-2,2-dimethyl-2H-1-benzopyran	3.241	0.244	3.754	0.265	3.792	0.224	0.008	0.051	0.855	0.863	+	
55	(R)-3-hydroxybutyrylcarnitine	5.148	0.152	5.454	0.096	5.426	0.046	0.007	0.051	0.949	0.944	+	
56	Sebacic acid	4.549	0.121	4.864	0.162	4.856	0.253	0.018	0.053	0.937	0.935	–	
57	Hypoxanthine	4.326	0.701	5.736	0.105	5.604	0.232	0.013	0.059	0.772	0.754	+	
58	Tyrosyl-Proline	3.637	0.311	4.267	0.331	4.117	0.272	0.018	0.067	0.883	0.852	+	
59	N-Monodesmethyl-rizatriptan	4.556	0.162	4.968	0.142	4.903	0.278	0.020	0.067	0.929	0.917	+	
60	3-hydroxyhexadecanoyl carnitine	5.761	0.103	5.948	0.131	5.985	0.151	0.023	0.075	0.963	0.969	+	
61	L-4-Chlorotryptophan	3.819	0.551	4.463	0.350	4.581	0.287	0.032	0.087	0.834	0.856	+	

HCMB, hypertensive intracerebral hemorrhage; HTN, hypertension; CON, control group; SD, standard deviation; FDR, false discovery rate. Mode: ion detection modes, including positive (+) and negative (-) ion modes.

The differences in the metabolites identified between the HCMB, HTN, and CON groups were sorted by FDR (corrected *P-*value) (**Tables 1**, **2**, **Figures 7B, C**). The top 10 significantly differentially expressed metabolites were 3-Formyl-6-hydroxyindole, atractyligenin, 2-keto-6-acetamidocaproate, LysoPC (*P-*18:0), PC (17:0/0:0), PS (O-20:0/0:0), PE (22:1/0:0), 2-O-methylcytosine, ganosporeric acid A, and PC (19:0/0:0). The top 10 differential metabolites with the most significantly decreased expression were 4, 11, 13, 15-Tetrahydroridentin B, guanidylic acid, 2,2’-(3- methylcyclohexane-1,1-diyl) diacetic acid, alpha-dihydroartemisinin, C75, 5-Nonyltetrahydro-2-oxo-3 furancarboxylic acid, 10-Oxo-11-octadecen-13-olide, 10,11-dihydro-20-trihydroxy-leukotriene B4, 10,11-dihydro-20-trihydroxy-leukotriene B4, and L-menthyl acetoacetate. To further confirm and test for more precise biomarkers, we constructed ROC curves for illness detection using SPSS (version 24.0). We selected metabolites with an AUC > 0.85 and a *P-*value < 0.01 (**Figure 8**, **Table 3**).

**Table 3:** Area below the curve for 14 differential metabolites.

Test variables	Area below the curve	Standard error	Asymptotic Significance	Approaching 95% confidence interval	
				Lower limit	Upper limit
3-Formyl-6-hydroxyindole	0.931	0.063	0.004	0.807	1.000
Isoputreanine	0.958	0.043	0.002	0.874	1.000
Citrulline	0.896	0.079	0.008	0.741	1.000
(+/-)-2-Methylthiazolidine	0.958	0.043	0.002	0.874	1.000
GlyGlyGlyVal	0.944	0.052	0.003	0.842	1.000
5,7-Megastigmadien-9-ol glucoside	0.917	0.068	0.005	0.783	1.000
Sphinganine phosphate	0.903	0.072	0.007	0.761	1.000
GammaGlutamylphenylalanine	0.903	0.072	0.007	0.761	1.000
Octanoylglucuronide	0.958	0.043	0.002	0.874	1.000
(+/-)-Hexanoylcarnitine	0.931	0.063	0.004	0.807	1.000
LysoPC(18:3(6Z,9Z,12Z))	0.931	0.063	0.004	0.807	1.000
S-Methyl hexanethioate	0.889	0.082	0.009	0.727	1.000
N-Monodesmethyl-rizatriptan	0.903	0.074	0.007	0.757	1.000
3-hydroxyhexadecanoyl carnitine	0.903	0.072	0.007	0.762	1.000

Among the 93 biomarkers screened above in the HCMB group with HTN and CON groups, eight metabolites were screened for upregulation: 3-formyl-6-hydroxyindole, isoputreanine, citrulline, (+/-)-2- methylthiazolidine, GlyGlyGlyVal, 5,7-megastigmadien-9-ol glucoside, sphi-nganine phosphate, GammaGlutamylphenylalanine. The six metabolites with decreased levels were octanoylglucuronide, (+/-)-hexanoylcarnitine, LysoPC (18:3(6Z,9Z,12Z), S-methyl hexanethioate, N-monodesmeth-ylrizatriptan, 3-hydroxyhexadecanoyl carnitine. These can be used as the most likely biological markers for the early diagnosis of HCMB.

### 3.4 Abnormal pathways were found to be involved in the HCMB process

**Figure 9:**
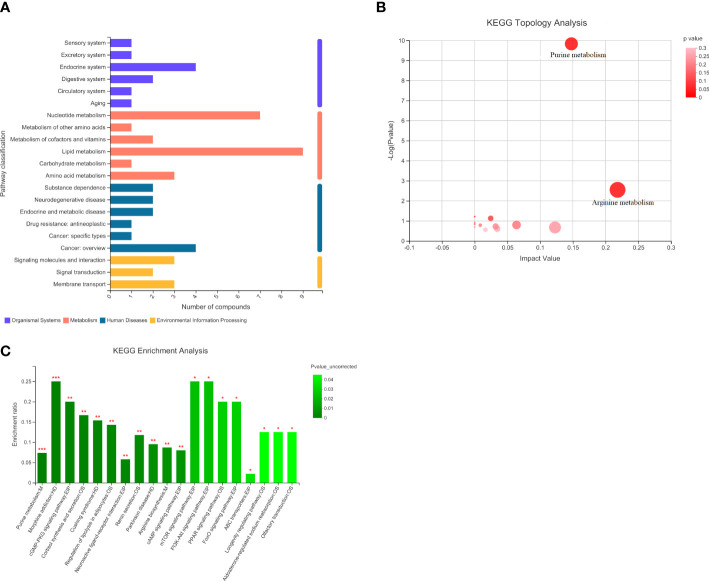
Statistical plots of differential metabolite classification, KEGG statistical plots and bubble plots of KEGG topology analysis. **(A)**. Categorical aggregation plot of 93 differential metabolites. Note: The vertical coordinate is the secondary classification category of the KEGG compound and the horizontal coordinate is the number of metabolites annotated to that classification. **(B)**. 93 differential metabolites KEGG topology analysis bubble map. Note: Each bubble in the graph represents a KEGG Pathway pathway; the horizontal axis indicates the relative importance of metabolites in the pathway in terms of Impact Value; the vertical axis indicates the enrichment significance of metabolite involvement in the pathway - log10(Pvalue); the size of the bubble represents the Impact Value value; the larger the bubble, the greater the importance of the pathway. **(C)**. KEGG statistical map of 93 differential metabolites. Note: The horizontal coordinate indicates the pathway name and the vertical coordinate indicates the enrichment rate, which indicates the ratio of the number of metabolites enriched in the pathway to the number of metabolites annotated to the pathway (Background number), the greater the ratio, the greater the enrichment. Column color gradients indicate the significance of enrichment, with the default color being darker, representing more significant enrichment for that KEGG term, where Pvalue or FDR < 0.001 is marked as ***, Pvalue or FDR < 0.01 is marked as **, and Pvalue or FDR < 0.05 is marked as *.

Comparing the HCMB metabolites to the CON and HTN groups, KEGG categorization showed that they primarily belonged to lipid and nucleotide metabolism (**Figure 9**). Citrulline and L-ornithine, which operate in the arginine metabolic route, are upregulated, whereas adenine, hypoxanthine, guanine, and adenylate, which act in the purine metabolic system, are downregulated, among the 93 differential metabolites in the CON group (**Table 4**). The primary pathways implicated in the pathophysiological mechanisms of HCMB were identified to be arginine metabolism (*P* = 0.0028) and purine metabolism (*P* = 2×10^-10^), according to KEGG pathway analysis combined with available literature (34–65).

**Table 4:** Metabolic pathway KEGG topology analysis.

	Pathway_ID	Pathway Desciption	Match_status	Num	Impact_value	Pvalue_uncorrected	Pvalue_corrected
1	map00220	Arginine biosynthesis	2|23	2	0.218235	0.002817	0.012208
2	map00230	Purine metabolism	7|81	6	0.147708	1.47E-10	9.56E-10
3	map00330	Arginine and proline metabolism	1|72	1	0.122702	0.211033	0.228619
4	map00760	Nicotinate and nicotinamide metabolism	1|50	1	0.063914	0.158686	0.257865
5	map00140	Steroid hormone biosynthesis	1|89	1	0.034327	0.24524	0.24524
6	map00240	Pyrimidine metabolism	1|62	1	0.031888	0.188424	0.244951
7	map00600	Sphingolipid metabolism	1|21	1	0.02439	0.073973	0.192329
8	map00520	Amino sugar and nucleotide sugar metabolism	1|107	1	0.016332	0.276107	0.256385
9	map00564	Glycerophospholipid metabolism	1|52	3	0.008447	0.163847	0.236668
10	map01240	Biosynthesis of cofactors	2|303	2	3.14E-05	0.205691	0.243089
11	map00830	Retinol metabolism	1|17	1	0	0.060747	0.197428
12	map00480	Glutathione metabolism	1|38	1	0	0.125929	0.272847
13	map00071	Fatty acid degradation	1|47	1	0	0.150788	0.280034

The third column is Pathway Description, which is the name of the pathway; the fourth column is Match_status, which indicates the participation of metabolites in the pathway. The sixth column is the Impact_value: the overall importance score of the pathway with a total score of 1; it is calculated based on the relative position of metabolites in the pathway; the seventh column is the enrichment significance P-value of metabolite participation in the pathway; the eighth column is the corrected P-value.

Guanine is the most significant potential biomarker of HCMB, as verified by ROC curves (AUC > 0.85, *P* < 0.05). In addition, among the differential metabolites between the HCMB and CON groups, adenine, hypoxanthine, guanine, allantoic acid, and adenosine were enriched in the purine metabolic pathway; imidazoleacetic acid, 1-methylhistamine, and iminomethyl-L-glutamic acid were enriched in the histidine metabolic pathway; ornithine, oxidized glutathione, and vitamin C were enriched in the glutathione metabolic pathway. Among the differential metabolites of HTN and CON, manganate, 3-dehydroquinolinate, and fructose-1-phosphate were enriched in the phenylalanine, complexine, and tryptophan metabolic pathways; citric acid and isocitric acid were enriched in the citric acid cycle; tyramine glucosinolate, inositol, and vitamin C were enriched in the vitamin C metabolic pathway; and imidazoleacetic acid, 1-methylhistamine, and iminomethyl-L-glutamate were enriched in histidine metabolism. Cysteine, oxidized glutathione, and vitamin C were enriched in the glutathione metabolic pathway.

Analysis of 93 differential metabolites in combination with the KEGG database identified nine metabolites annotated to the lipid metabolic pathway LysoPC (P-18:0), PC (17:0/0:0), sphingosine phosphate, 2-dodecatrienoic acid, ganosporic acid A, Palmitoyl-L-carnitine, Cortisol, LysoPC (18:3(6Z,9Z,12Z)), 13(S)-HODE, annotated to 7 metabolites of the nucleotide metabolic pathway (Adenine, Hypoxanthine, Thymine, Guanine, Adenosine Monophosphate), and Cortisol. In addition, 4 metabolites annotated to the endocrine system pathway (Adenosine, Cortisol, Adenosine monophosphate, 13(S)-HODE), 4 metabolites annotated to the cancer pathway (LysoPC (P-18:0), PC(17:0/0:0), LysoPC [18:3(6Z,9Z,12Z)], cortisol), 3 metabolites annotated to the membrane transport pathway (Adenosine, Ornithine, Inosine), 3 metabolites annotated to the signaling molecule and interaction pathway (Sphingosine phosphate, Adenosine, Cortisol) and some metabolites partially annotated in metabolism, biological systems, human diseases, environmental information processing, and other pathways. The analysis of KEGG pathway annotations showed that the top 20 metabolic pathways for 93 different metabolites were purine metabolism: M, morphine addiction: HD, cGMP-PKG signaling pathway: EIP, cortisol synthesis and secretion: OS, Cushing syndrome: HD, Regulation of lipolysis in adipocytes: OS, Neuroactive ligand-receptor interaction: EPI, Renin secretion: OS, Parkinson disease: HD, Arginine biosynthesis: M, cAMP signaling pathway: EPI, mTOR signaling pathway: EPI, PI3K-Akt signaling pathway: EPI, PPAR signaling pathway: OS, FoxO signaling pathway: EPI, ABC transporters: EPI, Longevity regulating pathway: OS, Aldosterone-regulated sodium reabsorption: OS, Olfactory transduction: OS (**Supplementary Figure 1**).

## 4 Discussion

This study of mouse serum untargeted metabolomics revealed 272 potential biomarkers of HCMB and revealed several of the most important metabolites. In addition, arginine and purine metabolism were revealed to be potential core metabolic pathways affecting this process.

Purines are by-products of nucleotide metabolism, primarily in the form of purine nucleotides, which play crucial roles in the production of coenzymes, regulation of metabolism, and energy supply (66, 67). Early purine elevation can help identify early stroke patients and predict their severity (34, 35). In addition, purines were discovered to be significantly raised (11.6 ± 8.9μm, while 7.1± 4.2μm in control) within 24 hours in patients with hemorrhagic stroke in prior clinical investigations. This may be related to the cellular mechanism by which ATP metabolism sustained in irreversibly damaged tissues leads to the cessation of purine production, whereas purine metabolism in damaged tissues continues without subsequent purine replenishment, resulting in a continuous increase in purine concentration until damaged tissues either repair or die (36). Purines are released as metabolites, such as adenine, guanine, inosine, and hypoxanthine in the salvage pathway of nucleic acid catabolism, which produces free purine bases (37). Therefore, hypoxanthine and adenine have been used to assess purine metabolism (38). The purine metabolic pathway revolves around the uric acid cycle, producing differential metabolites, including adenine, hypoxanthine, guanine, and adenosine, all of which are downregulated, which may be related to the high production of uric acid downstream and insufficient replenishment of hypoxanthine, guanine, etc., after damage to the body. In contrast, it has also been shown that uric acid accumulates in HCMB patients with increased serum homocysteine and is an important risk factor for HCMB (39). In an important study on the relationship between uric acid and cerebral hemorrhagic disease, serum uric acid levels were found to be strongly associated with a high incidence of HCMB in patients with hypertension (40). Restricted circulation of uric acid in the body may lead to a large accumulation of uric acid, which can exacerbate the occurrence of HCMB. Insufficient supplementation of raw materials such as guanosine and adenosine in this process can also positively inhibit purine metabolism. In contrast, the neuromodulator adenosine reduces acute neuroinflammatory injury following ICH by inhibiting the production of proinflammatory cytokines by activating cell surface-specific receptors, decreasing the production of tumor necrosis factor mRNA in the body, and reducing hematoma size and neutrophil infiltration after brain hemorrhage (41). Adenine, hypoxanthine, guanine, inosine, and adenosine also play key roles in stimulating presynaptic receptors on nerve endings and postsynaptic receptors on immune cells, regulating innate and adaptive immunity and promoting the release of inflammatory factors (42–46). In addition, dysregulation of organismal immunity and the release of inflammatory factors leads to an inflammatory cascade response that damages the blood-brain barrier, exacerbates cerebrovascular injury and lesions, and contributes to the development of HTN to HCMB (47, 48). Therefore, purine metabolism and its metabolites including adenine, hypoxanthine, and guanine, are involved in the pathophysiological process of HCMB by influencing the body’s immunity and modulating the inflammatory response, which may be an important pathway to prevent HTN from developing into HCMB.

Arginine metabolism is inextricably linked to the uric acid cycle, and the end-product of its metabolism is uric acid. The core metabolite of arginine metabolism, arginine, produces urea and L-ornithine under the combined action of arginase I and II in the cytoplasmic matrix and mitochondria, respectively (49–51). In addition, nitric oxide (NO) and citrulline can be synthesized by the action of l-arginine synthase (NOS) (52). Another core product, ornithine, can eventually be transformed into polyamines, proline, and glutamate through a series of biochemical reactions (52). The differential metabolites ornithine and citrulline were found to be significantly upregulated in the enriched arginine metabolic pathway in this study, whereas no metabolic differences were found for arginine, which was supported by previous differential cerebrospinal fluid (CSF) metabolites from 25 patients with cerebral hemorrhage (53, 54). Elevated levels of citrulline and unchanged l-arginine concentrations in the CSF indicate a significant increase in NOS activity during the early stages of ICH. Over time, asymmetric dimethylarginine gradually increases and begins to inhibit L-arginine synthase, reducing NO and citrulline synthesis, leading to a decrease in cerebrospinal fluid citrulline levels in the late stages of ICH (54). In the mouse model in this study, L-NAME was used to extensively inhibit NOS activity *in vivo* and *in vitro* (55, 56). Thus, the early appearance of a surge in citrulline in mice with cerebral hemorrhage implies that the NOS pathway plays an important role in the pathophysiological mechanisms of a cerebral hemorrhage. This pathway produces NO, which can regulate intravascular blood flow and oxygenation of brain tissue, modulate vascular tone and diastolic vascularity, regulate vascular permeability, and exert anti-platelet, anti-thrombotic, and anti-inflammatory effects, which are related to sympathetic regulation of the body (57, 58). NO produced by endothelial NOS in vascular endothelial cells diffuses into vascular smooth muscle cells and activates the soluble guanylyl cyclase-cGMP-dependent protein kinase pathway, which regulates vasodilation (59), a major mechanism that regulates blood pressure and affects vascular endothelial cell function and integrity. Inhibition of NOS by low doses of L-NAME promotes the elevation of blood pressure and exacerbates the effects of HTN on systemic vasculature and target organs (56, 60). Whereas, the increase in guanosine in the present study implies that it may be that sustained blood pressure elevation with NO negatively feeds back on sympathetic nerves, and the body compensates by promoting the NOS pathway to produce NO for vasodilation. However, it also suggests that the increased conversion of arginine to ornithine following inhibition of the NOS pathway may have exacerbated citrulline production. Arginase, the focal enzyme for the conversion of arginine to urea and ornithine, competes with NOS for the substrate L-arginine, leading to NOS uncoupling, massive production of NOS scavengers, superoxide, and peroxynitrite, inhibition of translation, and reduced stability of inducible NOS proteins (61–63). The massive synthesis of arginase inhibits the NOS pathway, decreases NO production, and exacerbates vascular endothelial injury and cerebrovascular disease (64). In addition to affecting the NOS pathway, arginase shifts the focus of arginine metabolism to ornithine, promoting the formation of ornithine to produce polyamines and L-proline, and to form large amounts of citrulline *via* the ornithine cycle in the presence of ornithine aminomethyltransferase. The above pathway was verified in previous high-salt-induced HTN rat studies, which showed that high-salt HTN rats have higher levels of arginase I and II in skeletal muscle small vessels and have an attenuated vasodilatory response to the endothelium-dependent dilator acetylcholine (65). When an arginase inhibitor was used, increased acetylcholine sensitivity and restored vasodilatory function were observed in the vessels of high-salt HTN rats, suggesting that the arginine-ornithine pathway enrichment is an important pathway in the mechanism of HCMB development, indicating that the value of this pathway warrants further study and may provide new insights into the intervention of HCMB.

Overall, this is the first metabolomic study focusing on the onset and development of HCMB in mice, using HTN and HCMB models to mimic the pathological processes of HTN and HCMB due to clinical blood pressure fluctuations. This overcomes the limitations of some previous CMB models, such as collagenase, laser, and inflammation-induced CMB models, which can only study pathophysiological processes following the onset of CMB (68). Using these models, specific biomarkers for HCMB that could be different from those in the hypertension model were discovered. We combined this model with untargeted metabolomics screening of HCMB biomarkers for early intervention, which may contribute to the precise prevention and early individualized treatment of HICH. Second, this study searched for potential metabolic pathways affecting HCMB using the HCMB model, which will provide new insights into the pathophysiological mechanisms of HCMB, assist in the diagnosis of HCMB, and provide some theoretical support for the prevention of HICH in hypertensive patients.

However, the application of this model has some limitations. First, the main experimental subjects of this model were C57BL6/J male mice, which cannot be used to explore the metabolic differences in female mice. Because of genetic background differences between mice and humans, there may be differences between their metabolic differences and clinical human metabolic changes. Therefore, translational research in this study remains to be validated through subsequent exploration. Furthermore, the un-targeted metabolomics study using the HCMB model to identify potential biomarkers of HCMB was collected from serum samples of three groups of mice at the same time point and did not observe a dynamic change in serum from healthy mice to mice with stable hypertension, fluctuating blood pressure, or CMB. It would be of greater clinical relevance to perform this dynamic study in serum to identify early warning biomarkers at different stages. However, observing serum metabolomic changes in mice at uniform time points could help to reduce metabolic differences due to normal aging physiological changes in mice. Moreover, the use of L-NAME to influence nitric oxide to induce intracerebral hemorrhage is indeed closely associated with arginine. Therefore, whether arginine can be identified as a potential metabolic pathway affecting cerebral microhemorrhage in hypertension needs to be confirmed in further studies. Finally, this study focused on metabolic changes in mouse serum. Although serum metabolic studies are more convenient for identifying early diagnostic markers of the disease and prevention, there are limitations in exploring the mechanism of HCMB development. Combining metabolomic analysis of brain tissue, CSF, and another multifaceted metabolism will accelerate the understanding of the mechanism of HTN in HCMB. Therefore, in the future, we need to extend the research framework by combining brain tissue, urine, feces, and other multi-dimensional products. To build a comprehensive system and find meaningful biomarkers to interpret the process of brain hemorrhage in healthy mice, which will help us achieve more accurate prevention and treatment measurements of ICH.

## 5 Conclusion

The metabolite profiles of HCMB mice were significantly different from those of HTN and normal mice, and 272 possible biomarkers could be utilized to indicate early HCMB, with citrulline being the most significant. A deeper understanding of the mechanisms of this influence needs to be explored further.

## Data availability statement

The original contributions presented in the study are included in the article/**Supplementary Material**. Further inquiries can be directed to the corresponding author.

## Ethics statement

This animal experiment was ethically reviewed by the Department of Laboratory Animal Science, Central South University.

## Author contributions

SW, XZ, and LZ designed the research and determined the structure of the manuscript. SW, XZ, and HZ were involved in the implementation of this study. SW, XZ, HZ, Y-Z, and T-XY selected the references and contributed to the writing. XZ and T-XY collected the data. SW, XZ, LC, YL, Y-Z, and LZ helped to analyze the results of this study. Y-Z and LZ contributed to the revision and finalization of the manuscript. All authors contributed to the article and approved the submitted version.
