# Supplementary figures and images for: Potential preventive markers in the intracerebral hemorrhage process are revealed by serum untargeted metabolomics in mice using hypertensive cerebral microbleeds
Source: Front Endocrinol (Lausanne). 2023 Apr 20;14:1084858. doi: 10.3389/fendo.2023.1084858 (PMC10159181; doi:10.3389/fendo.2023.1084858)

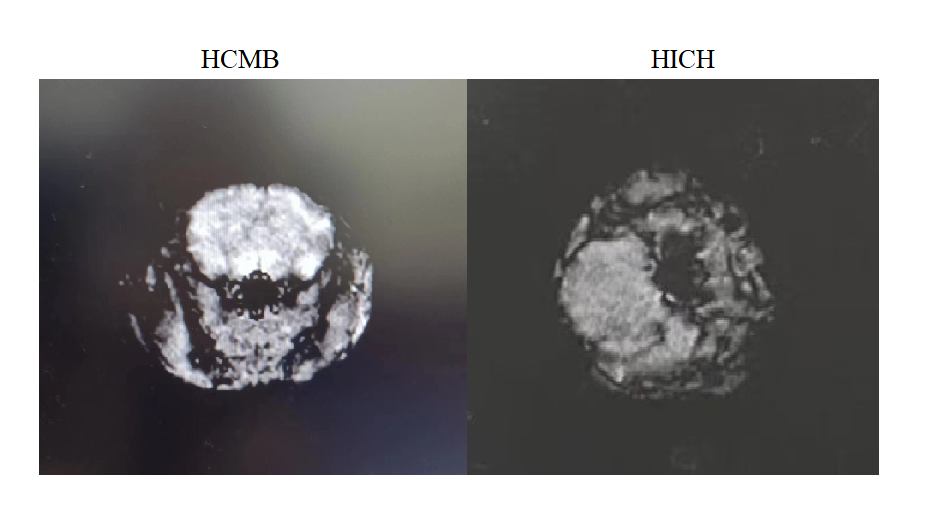

Supplement: Supplementary file 1 [file Image_1.tif]
