# Supplementary material for: Potential preventive markers in the intracerebral hemorrhage process are revealed by serum untargeted metabolomics in mice using hypertensive cerebral microbleeds
Source: Front Endocrinol (Lausanne). 2023 Apr 20;14:1084858. doi: 10.3389/fendo.2023.1084858 (PMC10159181; doi:10.3389/fendo.2023.1084858)
Supplement: Supplementary file 2 [file Table_1.docx]

**Supplementary Table 1: All analysis software used in this study and related information.**

| **Analysis items** | **Software** | **Versions** |
| --- | --- | --- |
| KEGG functional pathway | KEGG Pathway | Release 2017-05-01 |
| iPath metabolic pathway analysis | iPath3.0 | Version3.0 |
| Vip Analysis | ropls(R);scipy(Python) | Version1.6.2;Version1.0.0 |
| Differential metabolite analysis - multivariate statistics | ropls(R packages) | Version1.6.2 |
| KEGG compound classification | KEGG Compound | Release 2017-05-01 |
| Heatmap | scipy(Python) | Version1.0.0 |
| Cluster analysis | scipy(Python) | Version1.0.0 |
| Venn diagram | VennDiagram (R packages) | Version1.6.20 |
| PCA analysis | ropls(R packages) | Version1.6.2 |
| Relevance analysis | scipy(Python) | Version1.0.0 |
| KEGG pathway enrichment | scipy(Python) | Version1.0.0 |
